# Supplementary material for: Source-tracking ESBL-producing bacteria at the maternity ward of Mulago hospital, Uganda
Source: PLoS One. 2023 Jun 8;18(6):e0286955. doi: 10.1371/journal.pone.0286955 (PMC10249850; doi:10.1371/journal.pone.0286955)
Supplement: S1 Checklist — (DOCX) [file pone.0286955.s001.docx]

STROBE Statement—checklist of items that should be included in reports of observational studies

|  | Item No. | Recommendation | Page  No. | Relevant text from manuscript |
| --- | --- | --- | --- | --- |
| **Title and abstract** | 1 | (*a*) Indicate the study’s design with a commonly used term in the title or the abstract | **3** | **Lines 34-35** |
|  |  | (*b*) Provide in the abstract an informative and balanced summary of what was done and what was found | **3** | **Lines 36-55** |
| Introduction | | | |  |
| Background/rationale | 2 | Explain the scientific background and rationale for the investigation being reported | **4** | **Lines 58-82** |
| Objectives | 3 | State specific objectives, including any prespecified hypotheses | **6** | **Lines 101-105** |
| Methods | | | |  |
| Study design | 4 | Present key elements of study design early in the paper | **6** | **Lines 109, 119-133** |
| Setting | 5 | Describe the setting, locations, and relevant dates, including periods of recruitment, exposure, follow-up, and data collection | **6** | **Lines 109-117** |
| Participants | 6 | (*a*) *Cohort study*—Give the eligibility criteria, and the sources and methods of selection of participants. Describe methods of follow-up  *Case-control study*—Give the eligibility criteria, and the sources and methods of case ascertainment and control selection. Give the rationale for the choice of cases and controls  *Cross-sectional study*—Give the eligibility criteria, and the sources and methods of selection of participants | **7** | **Lines 118-133** |
|  |  | (*b*) *Cohort study*—For matched studies, give matching criteria and number of exposed and unexposed  *Case-control study*—For matched studies, give matching criteria and the number of controls per case |  |  |
| Variables | 7 | Clearly define all outcomes, exposures, predictors, potential confounders, and effect modifiers. Give diagnostic criteria, if applicable.  **Study outcome was ‘Detection of bacteria’ (page 7 line 137), resistance (page 8 line 150 &176, 190, 199, 226).**  **Predictors are described in Table 1 (Page 13, line 263)** | **7, 8**  **13** | **Line 137, 150&176,**  **190, 199.**  **Line 263** |
| Data sources/ measurement | 8* | For each variable of interest, give sources of data and details of methods of assessment (measurement). Describe comparability of assessment methods if there is more than one group  **The data collection is as described in the Methods section on page 6, lines 108-244. All data used here was primary data as collected using a questionnaire and biological samples as collected, the sample processing generated secondary and tertiary data i.e., susceptibility testing, PCR testing for genes as well as ESBL enzyme** | **6** | **Lines 108-244** |
| Bias | 9 | Describe any efforts to address potential sources of bias  **All pregnant women who met the selection criterion and gave consent were included, line 123 page 7** | **7** | **Line 123** |
| Study size | 10 | Explain how the study size was arrived at  **All pregnant women who met the criterion and gave consent were included line 123 page 7.** |  |  |

Continued on next page

| Quantitative variables | 11 | Explain how quantitative variables were handled in the analyses. If applicable, describe which groupings were chosen and why  **Analysis of similarity was done using clustering using phenotypic and genotypic molecular markers, this is a standard approach of investigating similarities and differences in bacterial strains. We use Ridom as the tool of choice. The bacterial strain comparisons are mapped to the hosts carrying them, i.e., Mother, Child and Hospital staff or environment to facilitate source tracking Page 12, Lines 237-244** | **12** | **Lines 237-244** |
| --- | --- | --- | --- | --- |
| Statistical methods | 12 | (*a*) Describe all statistical methods, including those used to control for confounding  **The variable groups (Mother, Child and health workers) sampled over time provide the comparators need, it is difficult to compare this group with any other ward, so a control is not appropriate. I suppose the health workers and the samples from the Ward environment could be viewed as controls - Page 13, Table 1.** | **13** |  |
|  |  | (*b*) Describe any methods used to examine subgroups and interactions  **Not applicable** |  |  |
|  |  | (*c*) Explain how missing data were addressed |  |  |
|  |  | (*d*) *Cohort study*—If applicable, explain how loss to follow-up was addressed  *Case-control study*—If applicable, explain how matching of cases and controls was addressed  *Cross-sectional study*—If applicable, describe analytical methods taking account of sampling strategy  **Fortunately, we do not have a lot of missing data in this study** |  |  |
|  |  | (*e*) Describe any sensitivity analyses  **No, this was not required** |  |  |
| Results | | | | |
| Participants | 13* | (a) Report numbers of individuals at each stage of study—eg numbers potentially eligible, examined for eligibility, confirmed eligible, included in the study, completing follow-up, and analysed  **137 mothers included and screened, bacteria recovered from 30 of them and their babies carried the bacteria of interest. The rest recovered from health workers and ward environments** |  |  |
|  |  | (b) Give reasons for non-participation at each stage  **All those how gave consent participated to the end** |  |  |
|  |  | (c) Consider use of a flow diagram |  |  |
| Descriptive data | 14* | (a) Give characteristics of study participants (eg demographic, clinical, social) and information on exposures and potential confounders  **See table 1 page 13 line 262** | **13** | **Line 262** |
|  |  | (b) Indicate number of participants with missing data for each variable of interest  **Enterobacteriaceae was recovered from 30 of 137 mothers followed, the all had metadata** |  |  |
|  |  | (c) *Cohort study*—Summarise follow-up time (eg, average and total amount) |  |  |
| Outcome data | 15* | *Cohort study*—Report numbers of outcome events or summary measures over time |  |  |
|  |  | *Case-control study—*Report numbers in each exposure category, or summary measures of exposure |  |  |
|  |  | *Cross-sectional study—*Report numbers of outcome events or summary measures  **30 of 137 mothers**  **30 of 137 babies see Tables 2- & 3-pages 13/14** |  |  |
| Main results | 16 | (*a*) Give unadjusted estimates and, if applicable, confounder-adjusted estimates and their precision (eg, 95% confidence interval). Make clear which confounders were adjusted for and why they were included  **We are not running any model here, we report proportions** |  |  |
|  |  | (*b*) Report category boundaries when continuous variables were categorized |  |  |
|  |  | (*c*) If relevant, consider translating estimates of relative risk into absolute risk for a meaningful time period |  |  |

Continued on next page

| Other analyses | 17 | Report other analyses done—eg analyses of subgroups and interactions, and sensitivity analyses |  |  |
| --- | --- | --- | --- | --- |
| Discussion | | | | |
| Key results | 18 | Summarise key results with reference to study objectives  **Page 18 lines 346- 355** | **18** | **Lines 346-355** |
| Limitations | 19 | Discuss limitations of the study, taking into account sources of potential bias or imprecision. Discuss both direction and magnitude of any potential bias  **Page 20 line 385-388** | **20** | **Line 385-388** |
| Interpretation | 20 | Give a cautious overall interpretation of results considering objectives, limitations, multiplicity of analyses, results from similar studies, and other relevant evidence  **Page 19: Figure 4 and lines 358 - 364** | **19** | **Lines 358-364** |
| Generalisability | 21 | Discuss the generalisability (external validity) of the study results  **Page 19 lines 366 to 383** | **19** | **Lines 366-383** |
| Other information | |  | | |
| Funding | 22 | Give the source of funding and the role of the funders for the present study and, if applicable, for the original study on which the present article is based  **Page 21 Lines 406-409** | **21** | **Lines 406-409** |

*Give information separately for cases and controls in case-control studies and, if applicable, for exposed and unexposed groups in cohort and cross-sectional studies.

**Note:** An Explanation and Elaboration article discusses each checklist item and gives methodological background and published examples of transparent reporting. The STROBE checklist is best used in conjunction with this article (freely available on the Web sites of PLoS Medicine at http://www.plosmedicine.org/, Annals of Internal Medicine at http://www.annals.org/, and Epidemiology at http://www.epidem.com/). Information on the STROBE Initiative is available at www.strobe-statement.org.
