# Supplementary material for: Source-tracking ESBL-producing bacteria at the maternity ward of Mulago hospital, Uganda
Source: PLoS One. 2023 Jun 8;18(6):e0286955. doi: 10.1371/journal.pone.0286955 (PMC10249850; doi:10.1371/journal.pone.0286955)
Supplement: S1 Table — (DOCX) [file pone.0286955.s002.docx]

**S 1 Table: Primer sequences used to PCR-amplify ESBL-encoding and carbapenemase-encoding genes**

| **Target** | **Primer sequence*** | **Amplicon size (bp)** | **Source** |
| --- | --- | --- | --- |
| *bla*_TEM_ | Forward: 5’-ATTCTTGAAGACGAAAGGGC-3’ Reverse: 5’-ACGCTCAGTGGAACGAAAAC-3’ | 1409 | [1] |
| *bla*_SHV_ | Forward: 5′-CAAAACGCCGGGTTATTC-3′ Reverse: 5′-TTAGCGTTGCCAGTGCT-3 | 937 | [2] |
| *bla*_CTX-M-U_ | Forward: 5’-CCGTCTAAGGCGATAAAC-3’ Reverse: 5’-TATCCGTACAAGGGAGTG-3’ | 157 | Inhouse |
| *bla*_CTXM-15_ | Forward: 5’-CCGTCTAAGGCGATAAAC-3’ Reverse: 5’-TATCCGTACAAGGGAGTC-3’ | 174 | Inhouse |
| *bla*_NDM_ | Forward: 5’-GGTTTGGCGATCTGGTTTTC-3’ Reverse: 5’-CGGAATGGCTCATCATCACGATC-3’ | 621 | [3] |
| *bla*_IMP_ | Forward: 5’-GAGGYGTTTATGTTCATAC-3’ Reverse: 5’-GTAMGTTTCAAGAGTGATGC-3’ | 587 | [4] |
| *bla*_VIM_ | Forward: 5’-GTTTGGTCGCATATCGCAAC-3’ Reverse: 5’-AATGCGCAGCACCAGGATAG-3’ | 382 | [5] |

*Primers were synthesized by Eurofins MWG Operon Inc., Austria

**References**

[1] Y. Sáenz *et al.*, “Mechanisms of Resistance in Multiple-Antibiotic-Resistant Escherichia coli Strains of Human, Animal, and Food Origins,” *Antimicrob. Agents Chemother.*, vol. 48, no. 10, pp. 3996–4001, Oct. 2004, doi: 10.1128/AAC.48.10.3996-4001.2004.

[2] D. M. Leinberger *et al.*, “Integrated Detection of Extended-Spectrum-Beta-Lactam Resistance by DNA Microarray-Based Genotyping of TEM, SHV, and CTX-M Genes,” *J. Clin. Microbiol.*, vol. 48, no. 2, pp. 460–471, Feb. 2010, doi: 10.1128/JCM.00765-09.

[3] L. Poirel, G. Revathi, S. Bernabeu, and P. Nordmann, “Detection of NDM-1-producing klebsiella pneumoniae in Kenya.,” *Antimicrob. Agents Chemother.*, vol. 55, no. 2, pp. 934–936, Feb. 2011.

[4] “Table 1.”, Accessed: Dec. 30, 2022. [Online]. Available: https://www.microbiologyresearch.org/content/jmm/10.1099/jmm.0.029868-0.t1

[5] F. Fallah, R. S. Borhan, and A. Hashemi, “Detection of bla(IMP) and bla(VIM) metallo-β-lactamases genes among Pseudomonas aeruginosa strains,” *Int. J. Burns Trauma*, vol. 3, no. 2, pp. 122–124, Apr. 2013.
