# Supplementary material for: Source-tracking ESBL-producing bacteria at the maternity ward of Mulago hospital, Uganda
Source: PLoS One. 2023 Jun 8;18(6):e0286955. doi: 10.1371/journal.pone.0286955 (PMC10249850; doi:10.1371/journal.pone.0286955)
Supplement: S2 Table — (DOCX) [file pone.0286955.s003.docx]

**S2 Table: Frequency and distribution of antibiotic resistance genetic elements among PCR-positive isolates (n=61)**

| **Species** | **Source** | **Antibiotic resistance genetic elements** | | | | | | |
| --- | --- | --- | --- | --- | --- | --- | --- | --- |
|  |  | *bla*_CTXM-U_ | *bla*_CTX-15_ | *bla*_TEM_ | *bla*_SHV_ | *bla*_NDM_ | *bla*_VIM_ | *bla*_IMP_ |
| *E. coli* | Wash sink | + | + | + | - | - | - | - |
| *E. coli* | Birth through CS/septic, cord | + | + | + | + | - | - | - |
| *E. coli* | Baby at discharge (armpit) | + | + | - | - | - | - | - |
| *E. coli* | Baby at discharge (groin) | + | + | + | - | - | - | - |
| *E. coli* | Mother | - | - | - | - | - | - | - |
| *E. coli* | Mother at discharge (groin) | + | + | + | - | - | - | - |
| *E. coli* | Mother at admission (nares) | + | + | - | - | - | - | - |
| *E. coli* | Mother at admission (groin) | + | + | - | - | - | - | - |
| *E. coli* | Mother at discharge (groin) | + | + | - | - | - | - | - |
| *E. coli* | Mother at discharge (groin) | + | + | - | - | - | - | - |
| *E. coli* | Health worker (finger tips) | - | - | - | - | + | + | - |
| *E. coli* | Health worker (finger tips) | + | + | + | - | - | - | - |
| *E. coli* | Health worker (finger tips) | + | + | + | + | + | - | + |
| *E. coli* | Health worker (finger tips) | + | + | + | + | - | + | + |
| *E. coli* | Health worker (finger tips) | - | - | - | - | - | - | - |
| *Kleb.** | Door | + | + | - | + | - | - | - |
| *Kleb.* | Door | + | + | - | + | - | - | - |
| *Kleb.* | Door | + | + | - | + | - | - | - |
| *Kleb.* | Drip stand | + | + | - | + | - | - | - |
| *Kleb.* | Baby | + | - | - | + | - | - | - |
| *Kleb.* | Baby | - | - | + | + | + | + | - |
| *Kleb.* | Baby | + | + | - | + | - | - | - |
| *Kleb.* | Baby | + | + | + | - | - | - | - |
| *Kleb.* | Baby (septic cord) | + | - | - | + | - | - | - |
| *Kleb.* | Baby at delivery (cord) | + | + | + | - | - | - | - |
| *Kleb.* | Baby at delivery (cord) | + | + | + | - | - | - | - |
| *Kleb.* | Baby at delivery (cord) | + | + | - | + | - | - | - |
| *Kleb.* | Baby at delivery (armpit) | + | + | + | + | - | - | - |
| *Kleb.* | Baby at discharge (groin) | + | + | + | + | - | - | - |
| *Kleb.* | Baby at discharge (groin) | + | + | - | + | - | - | - |
| *Kleb.* | Mother | + | - | - | + | - | - | - |
| *Kleb.* | Mother | + | + | - | + | - | - | - |
| *Kleb.* | Mother | + | + | + | + | - | - | - |
| *Kleb.* | Mother | + | + | - | + | - | - | - |
| *Kleb.* | Mother at discharge (armpit) | + | + | - | + | - | - | - |
| *Kleb.* | Mother at discharge (armpit) | + | + | - | - | - | - | - |
| *Kleb.* | Mother at discharge (armpit) | + | + | - | + | - | - | - |
| *Kleb.* | Mother at discharge (armpit) | + | + | + | + | - | + | - |
| *Kleb.* | Mother at discharge (groin) | + | - | + | + | - | - | - |
| *Kleb.* | Mother at discharge (groin) | + | + | - | - | - | - | - |
| *Kleb.* | Mother at discharge (groin) | + | + | - | - | - | - | - |
| *Kleb.* | Mother at admission (nares) | + | + | - | - | - | - | - |
| *Kleb.* | Mother at discharge (groin) | + | + | + | + | - | - | - |
| *Kleb.* | Mother at discharge (groin) | + | - | - | + | - | - | - |
| *Kleb.* | Health worker (Finger tips) | + | - | - | + | - | - | - |
| Enterobacter | Baby (septic cord) | + | + | + | - | - | - | - |
| Enterobacter | Baby at discharge (groin) | + | + | - | + | - | - | - |
| Enterobacter | Baby at discharge (groin) | + | + | + | - | - | - | - |
| Enterobacter | Baby at discharge (groin) | + | + | + | - | - | - | - |
| Enterobacter | Baby at discharge (groin) | + | + | + | - | - | - | - |
| Enterobacter | Baby at discharge (groin) | + | + | + | - | - | - | - |
| Enterobacter | Baby at discharge (groin) | + | + | + | - | - | - | - |
| Enterobacter | Baby at discharge (groin) | + | + | - | - | - | - | - |
| Enterobacter | Baby at discharge (groin) | + | + | + | + | - | - | - |
| Enterobacter | Baby at discharge (nares) | + | + | - | - | - | - | - |
| Enterobacter | Baby at discharge (nares) | + | + | + | + | - | - | - |
| Enterobacter | Mother at admission (groin) | + | + | - | - | - | - | - |
| Enterobacter | Mother at admission (groin) | + | + | - | - | - | - | - |
| Enterobacter | Mother at admission (nares) | + | + | - | - | - | - | - |
| Enterobacter | Mother at discharge (groin) | + | + | - | - | - | - | - |
| Enterobacter | Health worker (finger tips) | + | + | + | - | - | + | - |
|  | **Total, n** | **57** | **51** | **26** | **29** | **03** | **05** | **02** |

**Klebsiella pneumoniae*; CS, caesarian section; +, positive; -, negative

Colored cells depict isolates carrying multiple resistance genetic elements
