# Supplementary material for: Source-tracking ESBL-producing bacteria at the maternity ward of Mulago hospital, Uganda
Source: PLoS One. 2023 Jun 8;18(6):e0286955. doi: 10.1371/journal.pone.0286955 (PMC10249850; doi:10.1371/journal.pone.0286955)
Supplement: Attachment — Submitted filename: ESBL-producing bacteria Uganda review.docx [file pone.0286955.s004.docx]

This is a very informative and technical work. Few inputs made; work is good for publishing.

I appreciate the fact that you numbered your lines making reviewing this work a lot easier, though some numbered lines are blank I would use your numbering as is. Thank you

1. Line 34, page 9; please correct cross-section study to cross-sectional study
2. Lines 50 - 52, page 10; please review this statement and clarify
3. Lines 114 – 115, page 12; do you have access to more recent birth records? If yes, please use that unless you have a good reason for using these from 9 years ago.
4. Line 132, page 13; please do not abbreviate the word approximately
5. Line 137, page 14; please change carried out from to “carried out in “
6. Line 240, page 18; please use chi ^2^ or chi square
7. Lines 242 - 243, page 18; please change was to were
8. Include your keywords to the written manuscript
9. The omission of gloves used by health workers as part of samples to be taken was a huge oversight in this work nevertheless much work was put into this study and it is very technical and highly informative. The work itself has a lot of papers embedded
10. Justice was done to the title
